# Chronic Urticaria Treated with Sodium Nitrite

**Published:** 1898-05

**Authors:** 


### CHRONIC URTICARIA TREATED WITH SODIUM NITRITE.

In the *Pacific Record of Medicine and Surgery*, September, 1897, Dr. John P. Sawyer gives his experience in the treatment of chronic urticaria by the use of nitrite of sodium. The patient was a young lady who had been afflicted with urticaria during the summer season for many years. Many treatments had been tried, but nothing had brought relief. Not discovering any intrinsic cause for the condition, and regarding urticaria as an angio-neurosis, he prescribed one grain doses of sodium nitrite three times daily. The following day the patient experienced her first complete relief from the difficulty, and during the warm weather of the entire season she remained practically free from the disease. He offers no explanation for the relief afforded by the remedy than through its well-known effect upon the peripheral circulation.

Encouraged by success in this case, he prescribed it for another one with the same general effect. The withdrawal of the remedy, in each case, was followed by a reappearance of the urticaria; on readministering the drug the disease subsided. Although this remedy was used in only two cases, still the evidence is so strongly in its favor that it would deserve trial in this persistent and intensely annoying disorder.

